# Use of mixed methods research in intervention studies to increase young people’s interest in STEM: A systematic methodological review

**DOI:** 10.3389/fpsyg.2022.956300

**Published:** 2023-01-05

**Authors:** Sergi Fàbregues, Milagros Sáinz, María José Romano, Elsa Lucia Escalante-Barrios, Ahtisham Younas, Beatriz-Soledad López-Pérez

**Affiliations:** ^1^Department of Psychology and Education, Universitat Oberta de Catalunya, Barcelona, Spain; ^2^Internet Interdisciplinary Institute, Universitat Oberta de Catalunya, Barcelona, Spain; ^3^Department of Education, Universidad del Norte, Barranquilla, Colombia; ^4^Faculty of Nursing, Memorial University of Newfoundland, St. John’s, IL, Canada

**Keywords:** mixed methods research, qualitative research, STEM, intervention, methodological review

## Abstract

**Introduction:**

Mixed methods research intervention studies integrate quantitative evaluation approaches, such as randomized controlled trials and quasi-experimental designs, with qualitative research to evaluate the effectiveness, efficacy, or other results of an intervention or program. These types of studies, which have attracted growing attention in recent years, enhance the scope and rigor of the evaluation. While various frameworks that summarize the justifications for carrying out these types of studies and provide implementation guidance have been published in the last few years in the health sciences, we do not know whether such frameworks have been properly implemented in the social and educational sciences. This review examined the methodological features and reporting practices of mixed methods intervention studies aimed at increasing young people’s interest in STEM.

**Methods:**

A systematic search was carried out in APA PsycNET, ERIC, ProQuest, Scopus, and Web of Science, and a hand search in 20 journals. We included peer-reviewed English-language articles that reported intervention studies with a quantitative component measuring outcomes specific to increasing secondary school students’ interest in STEM fields, a qualitative component conducted before, during, or after the quantitative component, and evidence of integration of both components. Qualitative content analysis and ideal-type analysis were used to synthesize the findings.

**Results:**

We found 34 studies; the majority published in the last ten years. Several patterns of mixed methods application were described in these studies, illustrating the unique insights that can be gained by employing this methodology. The reporting quality of the included studies was generally adequate, especially regarding the justification for using a mixed methods intervention design and the integration of the quantitative and qualitative components. Nonetheless, a few reporting issues were observed, such as a lack of detail in the presentation of the mixed methods design, an inadequate description of the qualitative sampling and analysis techniques, and the absence of joint displays for representing integration.

**Discussion:**

Authors must pay attention to these issues to ensure that the insights obtained by the use of mixed methods research are effectively communicated.

## 1. Introduction

Mixed methods research (MMR) integrates quantitative and qualitative methods in a single study or sustained program of inquiry to generate a more complete understanding than is achievable with a single method (Fetters, 2020). The use of MMR has significantly increased in recent years and a variety of designs for its implementation have been proposed, each with its own aim, assumptions, procedures, and integration strategies (Creswell and Plano Clark, 2018). One of these is the MMR intervention design, which combines a quantitative evaluation design (i.e., randomized controlled trial [RCT], quasi-experimental design, non-experimental design) with qualitative research used to determine the effectiveness, efficacy, or other outcomes of an intervention or program. MMR intervention designs have received increasing attention in recent years. A growing number of methodological publications (Sandelowski, 1996; Lewin et al., 2009; O’Cathain et al., 2013; Zhang, 2014; Boeije et al., 2015; Grissmer, 2016; Johnson and Schoonenboom, 2016; Maher and Neale, 2019; Richards et al., 2019; Bouchard and Tulloch, 2020; Fetters and Molina-Azorin, 2020; Aschbrenner et al., 2022), including an entire textbook (O’Cathain, 2018), have described the ways in which designs of this type, when properly implemented, enhance the comprehensiveness, rigor, and efficiency of the intervention study.

One distinguishing feature of MMR intervention designs is their ability to transcend the limitations of RCTs in producing findings that are easily transferable to practice. Johnson and Schoonenboom (2016) summarized several of these limitations, including the inability to generalize the findings to other settings and populations and the fact that they are “performed in ideal circumstances” (p. 587), which might produce findings that might not be representative of the context of the intervention. Most of these limitations can be addressed by including qualitative research in the intervention study since this approach can help researchers to better understand the context and conditions surrounding the intervention, the contextual elements and causal mechanisms that generate the effects, how these mechanisms operate, and the differences between participants in the effects observed. By integrating qualitative research with a quantitative evaluation design, researchers can gather contextual and individual-specific knowledge about why, how, and under what conditions an intervention does or does not work. This more detailed understanding of the effects of the intervention will be critical in producing context-sensitive recommendations that can be implemented effectively in policy and practice. The qualitative phase, for example, might be used in implementation studies to assess the feasibility of an intervention and its implementation strategies, as well as to complete process and outcome evaluations (Cheng and Metcalfe, 2018; Landes et al., 2019).

Scholars working primarily in the health sciences have developed two main frameworks that describe reasons for using qualitative research in intervention studies. The first framework, the *temporal framework*, categorizes these reasons based on whether the qualitative component was implemented before, during, or following the intervention (Lewin et al., 2009; Johnson and Schoonenboom, 2016; Creswell and Plano Clark, 2018). For example, qualitative research undertaken before an intervention can aid researchers in evaluating the need for the intervention, generating hypotheses for testing in the quantitative part, and developing adequate outcome measures. The use of qualitative research during the intervention can aid researchers in determining the fidelity of the implementation methods, examining the perspectives of researchers carrying out the intervention, and identifying potential barriers and facilitators encountered by participants. After the intervention, researchers may use qualitative research to explain unexpected or non-significant quantitative findings, examine how the context may have influenced the findings, and identify research questions for further research. More recently, Maher and Neale (2019) proposed a variant of the temporal framework, called *temporal parallel purpose framework*, in which, maintaining the sequential logic of the previous frameworks, the authors classified the reasons according to whether they were related specifically to the intervention or the RCT. A second framework for using MMR intervention designs is the *Aspects of a Trial Framework*, which was developed from a review of 296 peer-reviewed health sciences articles published between 2008 and 2010 reporting qualitative research conducted with trials (O’Cathain et al., 2013). In that review, the authors were unable to use the temporal framework to code the reasons for doing qualitative research in the included studies because most of them did not provide the precise time period for the qualitative data collection. As a result, O’Cathain (2018), the principal investigator of the review, developed this second framework that classifies those reasons according to the following five main aspects of a clinical trial: (a) the intervention, (b) the trial design and conduct, (c) the outcomes, (d) the process and outcome measures used, and (e) the health condition addressed by the intervention. A summary of published examples of these two frameworks and its content can be found in Fetters and Molina-Azorin (2020).

Frameworks have been instrumental in illuminating the numerous possibilities that qualitative research can bring to the task of comprehensively and meaningfully evaluating interventions, particularly in the case of intervention researchers unfamiliar with MMR or skeptical of qualitative research. As a complement to more generic MMR methodological publications and textbooks, these frameworks have also served as practical guidelines for the design and implementation of MMR intervention studies. However, as described in several methodological reviews, predominantly in the health sciences, published empirical research consistently exhibits significant flaws in the reporting of design and implementation. Lewin et al. (2009) reviewed studies using qualitative research alongside randomized trials of complex healthcare interventions published during 2001 and 2003 and found that nearly half of them failed to report the qualitative sampling and analysis methods adequately, failed to justify the inclusion of a qualitative component, and failed to demonstrate integration. In the previously cited review by O’Cathain et al. (2013), the authors found that researchers frequently failed to explicitly acknowledge the contribution of the qualitative component to the study design and its added value. Similar findings were observed in a methodological review of the use and reporting quality of MMR in school-based obesity interventions by Brown et al. (2015), who reported that less than half of the studies justified the use of MMR and provided an adequate description of the MMR design. The authors also noted that, while most of the studies demonstrated evidence of integration of the quantitative and qualitative components, the reporting of this evidence frequently lacked detail and only a few studies described how it occurred. More recently, Thiessen et al. (2022) reviewed studies that combined RCTs and qualitative research in the field of oncology and concluded that the qualitative purpose was frequently not stated explicitly, the timing of the qualitative component within the overall design was frequently not reported, several aspects of the qualitative procedures were frequently not mentioned, and the integration of the quantitative and qualitative components was generally moderate. The methodological reporting flaws identified in these reviews warrant close examination because they may prevent researchers from fully communicating the unique insights afforded by an MMR approach.

While the literature on MMR intervention designs has contributed significantly to the advancement of this area of research practice, nearly all of these publications have been developed within the health sciences. To our knowledge, the only existing guidance on MMR intervention designs for researchers in the educational and social sciences was published by Grissmer (2016), who developed a guide that demonstrates the value of this type of design in evaluating educational and social interventions. This author asserted that the growing demand for MMR RCTs is a natural consequence of the current inadequacy of theories predicting social and educational outcomes. Since factors influencing outcomes of this type can be quite diverse due to the variety of the contexts in which interventions are implemented, existing theories may overlook some of these factors. Therefore, further developing these theories requires a more detailed and contextualized understanding of the multiple processes that contribute to the outcomes. According to Grissmer (2016), MMR intervention designs can contribute significantly to this understanding by clarifying the effects of context on intervention outcomes, elucidating why and how intervention effects occur, and explaining under what conditions the quantitative results are more reliable. Additionally, by generating this understanding, MMR designs of this type can be instrumental in elucidating the causal mechanisms underlying the long-term effects of the intervention (i.e., during a period after it is finalized).

The potential of MMR for generating contextualized knowledge is particularly relevant in the field of STEM (Science, Technology, Engineering, and Mathematics)-related interventions, as existing reviews indicate that a variety of contextual factors may contribute to differences in STEM education participation. For instance, van den Hurk et al. (2019) identified a number of factors that mediate and moderate participation in STEM education, some of which are context-specific, namely, the social context (i.e., educational policy, labor market/economy, and cultural environment/social views), the social environment (i.e., family and peers), and the school context (i.e., teaching pedagogy, school climate, and organization). Understanding these factors is crucial to developing successful interventions that would contribute to increasing interest in STEM programs and courses. Considering the limitations of quantitative research in properly capturing context, MMR intervention designs may enable researchers to achieve a more fine-grained and complete assessment of the range of contextual factors affecting the intervention outcomes. Additionally, this type of design can aid in the investigation of the long-term effects of STEM-related interventions, a subject that is particularly challenging to investigate due to the complexity of factors that act as long-term barriers to people becoming engaged in STEM (Prieto-Rodriguez et al., 2020).

The usefulness of MMR in evaluating STEM-related interventions was confirmed in a recent systematic review by Prieto-Rodriguez et al. (2020) of secondary school STEM interventions targeting girls. MMR was used in 19 of the 32 studies identified in that study. Despite the confirmed prevalence of these MMR studies, no reviews have been published that have systematically examined them. This omission is striking given the benefits of MMR intervention designs in developing context-specific knowledge that is easily transferable to policy and practice. Thus, an examination of the methodological features and reporting practices associated with this type of design is necessary to ascertain whether the added value of MMR is being realized in STEM-related interventions and whether the methodological limitations associated with MMR intervention designs in the health sciences also exist in this field. To address this need, our review aims to (1) characterize and describe the methodological features of MMR intervention studies intended to promote young people’s interest in STEM; and (2) to assess the reporting quality of these studies. In this review, we intend to contribute to the practice of STEM intervention research by describing how MMR can improve the effective and comprehensive evaluation of STEM interventions and by making recommendations for reporting MMR intervention studies in this field.

## 2. Methods

### 2.1. Design

A methodological review was carried out. According to Mbuagbaw et al. (2020), methodological reviews are studies that report “on the design, conduct, analysis, or reporting of primary or secondary research-related reports” (p. 1). By examining the methodological characteristics of a sample of studies within a field identified systematically, reviews of this type can assist researchers in expanding their methodological repertoire, identifying existing methodological gaps, and improving future research practice (Aguinis et al., 2020; Martin et al., 2020; Howell Smith and Shanahan Bazis, 2021). Methodological reviews are particularly important in MMR intervention research because some basic procedures of the methodology are still not being implemented properly, as revealed by a number of reviews (see Section “Introduction”). The studies included in this methodological review were identified through a scoping review of intervention studies aimed at increasing young people’s interest in STEM (Sáinz et al., 2022)—hereinafter referred to as the *original review*. Specifically, we focus here on the subsample of studies from the original review that used MMR. This review has been conducted and reported using the updated 2020 version of the Preferred Reporting Items for Systematic Reviews and Meta-Analyses (PRISMA) guidelines (Page et al., 2021). Since methodological reviews differ from conventional systematic reviews in their primary purpose and some of their procedures (Martin et al., 2020), only the PRISMA reporting criteria applicable to these reviews were used. Similarly, the protocol for this review was not registered due to the methodological nature of this study.

### 2.2. Eligibility criteria

To be included in the original review, publications had to: (a) report intervention studies aimed at increasing secondary school (i.e., in grades six and above according to the US educational system) students’ participation in STEM fields; (b) clearly describe the intervention’s objectives, participants, and context, as well as provide a concise description of its implementation; (c) evaluate the effectiveness of the intervention using a quantitative, qualitative, or MMR approach; and (d) be in English and published between 1998 and 2019. In the original review, non-empirical papers were excluded, including systematic reviews, editorials, and commentaries. In the methodological review, the same inclusion criteria as the original review were followed, except for the publication type, which was limited to peer-reviewed journal articles. In addition, studies included in the methodological review had to: (a) report quantitative research measuring outcomes specific to increasing secondary school students’ interest in STEM fields using a pre- post-measurement; (b) report qualitative research carried out before, during, or after the quantitative component; and (c) provide evidence of integration of the qualitative and quantitative components; include a description of where and how the integration was carried out; refer to an attempt at integrating methods, or else use words associated with integration.

### 2.3. Information sources and search strategy

In the original review, we searched the title and abstract of publications in English indexed between 1998 and 2019 in the following five databases: APA PsycNET, ERIC, ProQuest, Scopus, and Web of Science. The searches in all databases were carried out on February 5, 2020. We used search terms related to the following four concept areas: intervention (e.g., program*, interven*, course*), STEM studies and professions (e.g., STEM, math*, science*), outcomes (e.g., interest*, engag*, motivat*), and gender (e.g., gender, girl*, female*) (see Supplementary File 1 for the complete search query). The search strategy was developed in collaboration with an information scientist from the Universitat Oberta de Catalunya. In addition, we used three complementary search strategies to uncover relevant literature that database searches were unable to locate. First, we hand searched the following 20 journals publishing educational and behavioral STEM-related interventions: *American Psychologist*, *Annual Review of Psychology*, *Developmental Psychology*, *Educational Psychology Review*, *Educational Research, International Journal of Science Education*, *Journal of Applied Developmental Psychology*, *Journal of Educational Psychology*, *Journal of Experimental Child Psychology*, *Journal of Personality and Social Psychology, Personality and Social Psychology Bulletin*, *Perspectives on Psychological Science*, *Psychological Bulletin*, *Psychological Science*, *Psychology of Women Quarterly*, *Review of Educational Research, Science*, *Sex Roles*, *Social Psychological and Personality Science*, and *Social Science Quarterly*. Second, we reviewed the lists of publications of important authors in the field. Third, we scanned the references sections of key articles.

### 2.4. Selection process

The study selection was carried out in two phases. In the screening phase, two researchers independently screened the titles and abstracts of a random sample of 10% of the publications. Disagreements between the two reviewers were resolved through discussion with the involvement of a third reviewer when necessary. The remainder of the publications were divided between the two reviewers. In the eligibility phase, the two reviewers independently assessed their full texts and documented the reasons for exclusion. Disagreements in this phase were again resolved by consensus. EPPI-Reviewer was used in this phase for abstract and full text screening.

### 2.5. Data collection process and synthesis methods

We extracted and synthesized data from the studies included in this methodological review in three phases using qualitative content analysis (Schreier, 2012). In Phase 1, we read the full sample of the included studies to familiarize ourselves with the literature base we would synthesize. In Phase 2, we used the insights gathered during the familiarization phase to revise and update the extraction form we would use in the review. The extraction form, which the first author had previously used in two methodological reviews (Fàbregues et al., 2020, 2022), was guided by the literature on MMR intervention studies, the Good Reporting of Mixed Methods Studies (GRAMMS) guidelines (O’Cathain et al., 2008), and Fetters et al.’s (2013) typology of integration approaches (see Supplementary File 2 for the extraction form). In Phase 3, the first author used the extraction form from Phase 2 to extract passages from the included articles in Microsoft Excel. Data extraction was double-checked by three reviewers, and any disagreements were resolved by consensus. Phase 3 involved reviewing all the extracted passages and comparing them to identify patterns of similarity and differences in the methodological features of the articles. Literature summary tables were used in this phase (Younas and Ali, 2021). Additionally, we used Stapley et al.’s (2021) ideal-type analysis method to create a typology of the contributions of the qualitative component to generate evidence of effectiveness. Following the steps recommended by these authors, we analyzed the previously extracted passages reporting the rationale and insights for using an MMR approach and those providing evidence from the integration of the quantitative and qualitative components. These steps included the following: (a) familiarizing ourselves with the extracted passages from each of the included studies; (b) preparing a summary of these passages; (c) systematically comparing these summaries to form clusters (called “ideal types”) of similar studies based on the contribution of the qualitative component to the overall MMR design; (d) generating descriptions of the resulting ideal types and identifying studies that best represented each type; and (e) assessing the credibility of the typology by requesting an independent researcher to reclassify the studies into their ideal types, using the previously developed ideal-type descriptions. Steps b–d were carried out using MAXQDA version 2022.

## 3. Findings

**FIGURE 1:**
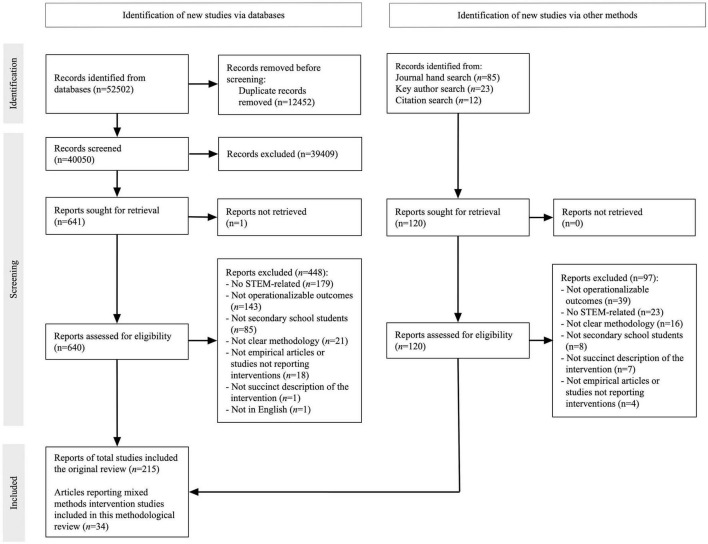
Preferred reporting items for systematic reviews and meta-analyses (PRISMA) flowchart.

The database and complementary searches yielded 40,170 records after removing duplicates. Two hundred fifteen studies were identified after assessing eligibility. Of these, 34 met the inclusion criteria for this review (see Supplementary File 3 for a list of included studies). The PRISMA flowchart of the review process is in Figure 1, along with the reasons for excluding publications in the eligibility phase.

### 3.1. General characteristics of the included studies

The complete characteristics of the 34 MMR intervention studies included in the review are shown in Supplementary File 4. More than three quarters (*n* = 28) of the studies were published in the last 10 years, of which 19 were published in 2016–2020 and nine in 2011–2015. Six studies were published before 2011, equally distributed in the periods 2000–2005 (*n* = 3) and 2006–2010 (*n* = 3). The studies were published in general (*n* = 4) and field-specific (*n* = 18) education journals, including those devoted to science education (*n* = 7), educational technology (*n* = 6), and other subfields (*n* = 5). Only four studies were published in non-educational journals. In 23 studies, the intervention took place in the United States, followed by three in the United Kingdom, two in Israel, and one in several countries, including Australia, Austria, Bolivia, Greece, Panama, South Africa, Spain, and Taiwan. Most of the interventions aimed to increase participants’ interest in STEM (*n* = 12) and science (*n* = 11) fields, whereas fewer aimed to increase their interest in technology (*n* = 8) and STEAM (*n* = 3) fields. Motivation was one of the outcome measures in nearly all studies (*n* = 33), while achievement was measured in more than half of the studies (*n* = 16). Gender stereotypes (*n* = 8), identity (*n* = 6), emotional outcomes (*n* = 2), and academic choices (*n* = 1) were also addressed to a lesser extent. In 20 studies, students were both girls and boys, whereas, in 14 studies, participants were solely girls. More than half of the studies (*n* = 19) made explicit reference to a theoretical framework, either from psychology (e.g., expectancy value theory of motivation, social learning theory) or education (e.g., constructivist and learning-related theories).

Consistent with our inclusion criteria, all the included studies (*n* = 34) used the quantitative component to measure intervention effectiveness outcomes, while two of them also used this component to assess the acceptability of the intervention. Qualitative methods were also employed in the full sample of 34 studies to determine the perceived effectiveness of the intervention. Several studies replicated the pre-post quantitative data collection procedures in the qualitative component to assess changes in participants’ views of intervention outcomes. For instance, Hughes et al. (2013) included an open-ended question on pre- and post-surveys to compare participants’ levels of interest in STEM, self-concept related to STEM, and their perceptions of scientists before and after the intervention. In other cases, participants’ views on the intervention effects were assessed retrospectively. For example, Aguilera and Perales-Palacios (2020) utilized a semi-structured interview with the participant teacher at the end of the intervention to elicit his views “on the effects of the intervention on student attitudes toward science and academic performance.” Likewise, Archer et al. (2014) carried out focus groups with female students at the end of the intervention to explore their perceptions of whether “they had learnt anything” and if “they felt their attitudes to STEM careers had changed.” In 11 studies, qualitative methods were used to evaluate the acceptability of the intervention, including “if they [the participants] had enjoyed participating in the [intervention]” (Ferreira, 2002), “which of the activities they liked and disliked” (Fabian and Topping, 2019), and “suggestions for improving [the intervention]” (Marino et al., 2013). Feasibility and fidelity were two other intervention domains examined in the qualitative component, each in three studies.

Half of the studies (*n* = 17) used the term “mixed methods” to describe the type of methods used, while the other half did not use any term. Only nine studies cited a methodological publication on MMR to justify this approach or explain its procedures. Five textbooks by Prof. John W. Creswell were among the six most cited publications, followed by the works of other influential MMR authors, such as Alan Bryman, Jennifer Greene, and Sharlene Hesse-Biber (see Supplementary File 5 for a list of the key MMR publications cited by the included studies). None of these nine studies citing MMR methodological publications cited a publication explicitly focused on MMR intervention designs.

### 3.2. Methodological characteristics and reporting quality

Table 1 illustrates the reporting quality of the 34 studies in terms of their compliance with each of the six GRAMMS guidelines. Supplementary File 6 gives further information regarding the methodological aspects of the studies.

**TABLE 1:** Reporting quality of the included studies in the review based on an adapted version of the good reporting of mixed methods studies (GRAMMS) guidelines (*n* = 34).

Guideline	Yes^a^	Yes, but^a^	No^a^
Describes the justification for using MMR to the research question	19	15	0
Describes the MMR design in terms of the purpose, priority, and sequence of methods	4	29	1
Describes each method in terms of sampling, data collection, and analysis	19	15	0
Reports evidence of integration^b^	30	1	3
Describes any limitation of one method associated with the presence of the other method	0	0	34
Describes any insights gained from mixing or integrating methods	6	3	25

MMR, mixed methods research.

^a^These categories are described in detail in Supplementary File 7.

^b^For the purpose of this study, the authors reformulated the original guideline number 4.

#### 3.2.1. Rationale for using MMR

Despite the advantages of MMR over mono-method research for achieving additional insights into the studied phenomenon, it may not always be the appropriate option for addressing particular types of evaluations. Certain research questions may be better addressed using a quantitative or qualitative approach alone. For this reason, researchers must present a persuasive case for why MMR is the best approach for carrying out a particular intervention study. All 34 studies included in the review provided either an explicit or implicit rationale for choosing an MMR design to carry out the intervention study. This rationale was articulated explicitly in 19 studies, with most of these citing or quoting MMR methodological references to support the use of this methodology. Some of these studies reported rationales commonly cited in MMR textbooks, such as taking “advantage of the virtues of the quantitative and qualitative methodologies, compensating the weaknesses of one with the strengths of the other” (Aguilera and Perales-Palacios, 2020) or “bringing to light as many aspects as possible of students’ activities in class” (Barak and Asad, 2012). In the remaining 15 studies, although this rationale was not explicitly stated, both quantitative and qualitative objectives were described in detail, allowing the reader to infer why an attempt was made to integrate both methodologies. Regardless of whether the rationales were explicitly or implicitly stated, in all the included studies, they were strongly tied to the ways in which the qualitative component complemented, strengthened, or supported the generation of evidence of effectiveness by the quantitative component. Examining these rationales, together with the integration outcomes and the insights gained from the use of MMR described in each article, we developed a typology of rationales for using qualitative research to generate additional evidence of effectiveness within the MMR study. Table 2 shows these rationales, along with a description and an example for each. As shown in the table, the studies in our sample most frequently used the qualitative component to provide confidence in the integrity of the quantitative outcomes (*n* = 18); to enhance, augment, explain, or illustrate the quantitative outcomes (*n* = 14), to assist in identifying intervention components that may have influenced the quantitative outcomes (*n* = 8), and to help explain heterogeneity within the participants’ responses to outcomes (*n* = 6).

**TABLE 2:** Rationales for using qualitative research to generating additional evidence of effectiveness.

Rationale	Description	Example
Corroborate the QUAN findings (*n* = 18)	QUAL provides confidence about the integrity of the QUAN outcomes	“The interviews (…) reinforce the trends we detected in the quantitative assessment; student quotes revealed positive impacts of the program on scientific self-efficacy, interest in pursuing STEM in the future, as well as the importance of dissemination in shaping their identity as a scientist” (Broder et al., 2019)
Determine why and how the outcomes occurred (*n* = 14)	QUAL findings are used to enhance, augment, explain, or illustrate the QUAN outcomes	“Table 3 above shows that both career interest (*Z* = 4.70, *p* < 0.001) and intrinsic interest (*Z* = 3.41, *p* = 0.001) reported significantly higher scores on the post-test after our 1-week camp (…). In the interviews, all campers mentioned at least one reason why the camp contributed to their interest in programming” (Clarke-Midura et al., 2019)
Identify intervention strengths and weaknesses (*n* = 8)	QUAL findings assist in identifying intervention components that may have affected the QUAN outcomes	“Over the course of the day, the girls also came to see science as more interesting and enjoyable (i.e., as having higher intrinsic value); qualitative data indicated that this was due to the variety of topics covered and the hands-on activities in which they participated” (Skipper and de Carvalho, 2019)
Explain differences in effectiveness within the sample (*n* = 6)	QUAL helps explain or better identify variability in participant responses to QUAN outcomes	“Male students consistently rated the activities higher than the girls. Their VMT scores were also significantly higher. In the student interviews, female students provided fewer positive responses about the intervention than male students. One possible reason for this is the nature of the paired work. Some girls that were paired with boys did not manage to work particularly well with their partner as the boys tended to take control of the tablet. This hesitation to work with the opposite sex was mentioned several times in the interviews” (Fabian and Topping, 2019)
Identify additional intervention benefits (*n* = 2)	QUAL helps identify additional benefits in addition to those represented by positive QUAN outcomes	“In addition to our two primary research questions, we also explored any additional benefits for students from participating in EPICC. The follow-up [qualitative] surveys pointed to a number of lasting impacts on participants. For instance, students reported that their experience in the service-learning project helped them feel a sense of contribution and connectedness to other people, as well as gratitude” (Collins et al., 2020)
Overcome study weaknesses by utilizing multiple sources of evidence (*n* = 1)	QUAL is added to other sources of QUAN data to compensate for the study’s inherent limitations (e.g., small sample of intervention participants)	“(…) the small number of students who participated in this exploratory work prohibits generalization. Nevertheless, questionnaire and interview data, personal observations both from teachers and researcher (…) and the development of text quality all indicate that the concept presented here may contribute to a positive interest development amongst high-school students with respect to NaSc” (Simon et al., 2016)
Improve confidence in the use of QUAN measures (*n* = 1)	QUAL and QUAN data are triangulated to obtain confidence in the application of QUAN measures when their validity and reliability have not yet been tested	“(…) the middle school surveys have not been administered to enough participants to be declared reliable and valid—this process is currently occurring. As a result, we chose to do our own reliability tests and use the quantitative data as a source of triangulation for the qualitative data (…) The qualitative codes matched the quantitative categories in 90% of the instances. With this triangulation using our qualitative data, we were confident in our decision to use these measures” (Hughes et al., 2013)
Reveal conflicting findings (*n* = 1)	QUAL findings conflict with QUAN outcomes, demonstrating the need for further inquiry	“This construct [understanding of computing jobs] was measured using two sources of data to determine whether students’ understanding changed over time, and they revealed conflicting findings” (Denner et al., 2012)

QUAN, quantitative; QUAL, qualitative.

#### 3.2.2. MMR design

Mixed methods research studies should report the elements of their procedural design, including the sequencing of the quantitative and qualitative components (i.e., the timing of their execution) and whether one had priority over the other. Several typologies of MMR designs have been published, the most well-known of these developed and refined over the past 20 years by Creswell et al. (2003) and Creswell and Plano Clark, 2007, 2011, 2018. Only four of the 34 studies included in this review provided a detailed explanation of the MMR design employed (Hur et al., 2017; Broder et al., 2019; Aguilera and Perales-Palacios, 2020; Chapman et al., 2020). All four of these cited one of Creswell’s typologies of MMR designs to support the assertion that they used a convergent design. Convergent designs involve the separate collection of quantitative and qualitative data, followed by their integration for comparison or combination. In accordance with this approach, Hur et al. (2017) collected two distinct databases (i.e., quantitative data using surveys and qualitative data using focus groups, participant observation, and open-ended questions), each tentatively having equal priority, and integrated them during the analysis and interpretation phases to enhance the trustworthiness of the study. In one of these four studies (Broder et al., 2019), the design was incorrectly labeled. While the authors claimed to have used a sequential design, they actually employed a convergent design because one database did not inform the other, as is the case with sequential designs. Twenty-nine studies did not specify the type of MMR design used, but they did describe the sequence of the components, namely the time at which qualitative approaches were utilized within the MMR intervention design. Only one study did not indicate the MMR design type as well as the sequencing and priority of the components.

#### 3.2.3. Quantitative and qualitative components

In addition to the specific MMR features, an MMR study must include quantitative and qualitative components that are elaborated with technical competence and reported transparently. While 19 of the studies reported in detail all quantitative and qualitative procedures, including sampling, data collection, and analysis, 15 studies failed to accurately report at least two of these procedures. In those studies, the authors frequently did not describe the methods used to analyze the qualitative data (*n* = 10) and/or the criteria that informed the selection of the qualitative sample (*n* = 8). For instance, while some of them described the characteristics of the participants in the qualitative component, they did not indicate why and how the researchers selected that particular group of participants over others.

In the quantitative component, single-group pre- and post-treatment designs (*n* = 25) were employed the most often, while other types of designs, such as multiple-group pre- and post-treatment designs (*n* = 5) and RCTs (*n* = 4), were employed much less frequently. In the qualitative component, only one study reported the qualitative design used, and this was ethnography. In all the studies, questionnaires were the primary quantitative data collection method (*n* = 34), accompanied in some cases by achievement exams (*n* = 5), content knowledge tests (*n* = 3), observation checklists (*n* = 2), quantitative content analysis (*n* = 2), and other methods (*n* = 3). In the qualitative component, the methods used were interviews (*n* = 23), open-ended questions (*n* = 14), focus groups (*n* = 11), observations (*n* = 8), and other methods (*n* = 5). The use of multiple data collection methods was marginally less prevalent in the quantitative component (*n* = 14) than in the qualitative component (*n* = 16). Lastly, qualitative data were obtained at various different times throughout each study, specifically in 12 studies before the intervention, in 10 studies during the intervention, in 30 studies immediately after finishing the intervention, and in six studies a few months after the intervention was completed. In only one study this information was not clear.

#### 3.2.4. Integration

In an MMR intervention study, integration involves mixing quantitative and qualitative components in one or more phases of a study to generate insights that lead to a more precise and exhaustive evaluation of the intervention. To effectively communicate these insights, researchers must provide a precise description of the integration outcomes and the resulting knowledge. Thirty of the 34 studies included in our review provided explicit evidence of integration; three did not provide any evidence; and in one study, the insights gained from integration could be inferred.

In the studies that provided explicit or partial integration reporting, we coded how integration was carried out using Fetters et al.’s (2013) typology of integration strategies. These authors explained that integration can occur through merging (when the two types of data or findings are brought together for comparison or analysis), building (when the findings from one component are used to define the data collection strategy of the other component) and connecting (when the findings from one component are used to define the sampling strategy of the other component). Thirty-one studies integrated through merging, two studies integrated through building and only one integrated through connecting. When merging was employed, the authors described the relationship between the quantitative and qualitative findings, including whether one form of data confirmed, expanded, or contradicted the findings of the other type. For instance, in a study evaluating two informal science programs, Hughes et al. (2013) described how the quantitative *t*-test findings indicating positive changes in the participants’ STEM identity confirmed the interview findings, which “also provided qualitative evidence of their [the participants’] improved trajectories.” Conversely, in an evaluation study of a project-service learning curriculum for high school students, Ruth et al. (2019) explained how the quantitative findings contradicted the qualitative ones. While, according to the quantitative findings, the project under evaluation was “not creating much change in the skills domains that could support any students’, including URM (historically underrepresented minority) and female students’, pathways into Engineering/STEM,” the qualitative ethnographic data indicated it was “positively impacting URM and female students in particular, and in ways that are meaningful and could potentially orient them toward STEM.”

In the two studies that integrated through building, the authors used the findings from one component to inform the data collection approach of the other component. Based on the quantitative data, Magerko et al. (2016) concluded that the learning module EarSketch was effective in enhancing students’ computing content knowledge and intent to persist in computing. To fully understand the success of this module, the authors carried out two focus groups using an interview guide based on the main conclusions from the quantitative findings. Lastly, in the study by Hughes et al. (2013) cited above, the authors integrated through connecting by selecting the participants in the interviews based on their scores in the quantitative measures (e.g., STEM self-concept, parental education, and exposure to STEM role models) to build a heterogeneous qualitative sample.

Using the same typology described by Fetters et al. (2013), we classified the ways in which integration was reported. All studies that provided explicit or implicit evidence of integration (*n* = 31) used a narrative approach to report the relationship between the two types of data. In these studies, this relationship was frequently explained verbally in both the results and discussion sections (*n* = 16), and less frequently in the results (*n* = 8) or discussion (*n* = 7) sections alone. Overall, the authors devoted substantial space to elucidating the interrelationships between the different quantitative and qualitative findings, thereby contributing to a more robust reporting of the integration outcomes. No studies used tables, diagrams, matrices, or figures to visually integrate the findings in the form of joint displays.

#### 3.2.5. Limitations and insights

No limitations as a result of using one methodological approach in conjunction with the other were reported in any of the articles. Furthermore, only six publications offered a clear description of the added value gained by utilizing an MMR approach in the discussion or conclusions sections. In these studies, authors declared that MMR allowed them to “gain invaluable insights on the effects of the games that could not have been discovered only through quantitative tests” (Kebritchi et al., 2010), or provide “different levels of granularity in the investigation of the effects” of the intervention (Fabian and Topping, 2019), amongst others. In three studies, the added value was not explicitly stated, but could be inferred.

## 4. Discussion

### 4.1. Summary of main findings

This review is, to the best of our knowledge, the first work to examine the use of MMR intervention designs in education and the social sciences. It is of particular interest given that most of the methodological publications about MMR intervention designs deal with the health sciences. As well as providing guidance for implementing designs of this type, these publications have shown that MMR intervention designs are becoming more popular owing to their usefulness in expanding the scope and strengthening the credibility of intervention evaluations in the health sciences. In this review, we examine whether such prevalence and advantages are also present in MMR intervention studies in the social and educational sciences in light of recent claims that such designs provide essential context and population-specific information for these interventions (Grissmer, 2016).

Our findings show an increase in the publication of MMR studies of interventions aimed at stimulating young people’s interest in STEM, with more than half of these studies having been published since 2016. This conclusion is congruent with the findings of a recent review of MMR interventions in emotional and behavioral disorders by Fàbregues et al. (2022), which identified a similar increase in the number of MMR intervention studies in that field. Moreover, our findings show that the incorporation of qualitative approaches into quantitative intervention designs was especially helpful in the study of interventions aimed at enhancing young people’s engagement in STEM, and particularly in elucidating how, under what conditions, and for what types of populations such interventions were successful or unsuccessful. For instance, in a study analyzing the success of a computer science programming summer camp for middle school kids, Clarke-Midura et al. (2019) used interviews with camp attendees to gain valuable insights on why and how the positive quantitative outcomes occurred. In qualitative interviews, the authors were able to discover numerous social elements that influenced participants’ positive shift in interest in STEM, including the opportunity to show their parents the apps they had built, the ability to provide and receive advice, and/or the availability of mentors. In another study assessing the impact of mobile technology on success in mathematics, Fabian and Topping (2019) were able to qualitatively discover that the intervention effects varied by gender, a conclusion that could not have been reached using purely quantitative methods. The authors determined, through student interviews, that male students viewed the activities more favorably than female students because some female students were matched with males who frequently assumed full control of the tablet. Furthermore, in several studies, a qualitative component was included for triangulation purposes to bolster the quantitative findings. In addition to quantitative measures of self-efficacy and interest in STEM subjects, Broder et al. (2019) used data from qualitative interviews to confirm the beneficial patterns revealed in the quantitative component. These trends suggest that the authors of the included studies were aware of the benefits of MMR intervention designs and employed them for the reasons cited in the health sciences frameworks described above. However, none of the included studies cited these frameworks or any methodological work on the combination of qualitative research and quantitative trials. This conclusion is relevant because it implies that the use of MMR in intervention designs was driven more by the intention to answer specific research objectives than by the literature.

In terms of reporting the MMR components, the 34 studies included in our study displayed a generally high level of quality. All the studies provided a justification for using an MMR intervention design, and more than half of them did so explicitly. This finding contrasts with previous reviews of MMR intervention designs in the health and behavioral sciences (Lewin et al., 2009; O’Cathain et al., 2013; Broder et al., 2019), in which the rationale for incorporating a qualitative component into a quantitative intervention design was either not provided or not detailed enough. Clarity in the reporting of the justification for using MMR was enhanced by the fact that, in most studies, the reason for including a qualitative component was explicitly mentioned, allowing the reader to understand how the qualitative aim interacted with the quantitative purpose. Integration of the quantitative and qualitative elements was also well-reported. Nearly all the studies integrated by merging, and the majority of these clearly reported the integration outcomes. Often, the reporting of integration was enhanced by extensively describing the outcomes in several subsections of the studies, particularly in both the findings and discussion sections. This form of reporting is consistent with Bazeley’s (2015) suggestion that integration reporting should not be restricted to the discussion section in order to maximize the integrative potential of MMR. According to this author, a greater emphasis on making explicit the linkages between the findings of both components throughout the entire manuscript, as was the case in several of the studies reviewed, could certainly result in a better integration. In addition, our review findings contradict the results of earlier reviews of MMR intervention designs, in which studies modestly reported integration. For instance, in a recent review of these types of designs in oncology, Thiessen et al. (2022) concluded that integration was often “limited to a brief statement regarding how a study conclusion was supported by both the qualitative and RCT data.” Not often was this the case among the studies analyzed in this review.

However, despite the generally good reporting quality of the reviewed studies, we nevertheless found three main issues. First, very few studies provided an accurate description of the design used, even though in most of them it was possible to identify the relative timing of the qualitative and quantitative components, a finding that contrasts with the findings from O’Cathain et al. (2013) and Thiessen et al.’s (2022) reviews of qualitative research utilized with RCTs in the health sciences. Second, even though the reporting of the methods followed in the quantitative component was generally detailed, the description of several qualitative procedures, particularly the qualitative sampling and analysis, lacked the same level of detail. Similar reporting issues with these two qualitative aspects have been identified in the reviews by Lewin et al. (2009) and Fàbregues et al. (2022). Third, no studies integrated both types of data using joint displays, which are visual tools based on tables or figures for performing and representing integration in MMR more clearly (Guetterman et al., 2015, 2021). Previous methodological reviews of MMR intervention designs have also found that none or very few of the included studies used displays of this type (Fàbregues et al., 2022).

### 4.2. Implications for the reporting of MMR studies for evaluating STEM-related interventions

Based on the previously observed inconsistencies in the reporting of MMR in the field of STEM-related interventions, we can draw three implications for authors of studies of this type. First, authors must describe the type of MMR design used in their studies, either by citing one of the existing typologies of MMR designs or by providing details of the purpose of their design, the timing of the quantitative and qualitative components, and the points of mixing between these components throughout the study. This can be achieved, for instance, through the use of procedural diagrams. According to Creswell and Plano Clark (2018), procedural diagrams can facilitate the intuitive representation of the MMR study features, thereby making it easier for the readers to “convey the complexity of mixed methods designs.” This is particularly relevant for MMR intervention studies due to the greater degree of complexity than other MMR studies using core designs (i.e., convergent, explanatory sequential, and exploratory sequential designs). Second, authors must provide transparent and accurate reporting of qualitative methods, including qualitative sampling and data analysis procedures. Even though the quantitative component tends to play a prominent role in these types of designs, this does not imply that the qualitative component should not adhere to adequate reporting standards. Third, in addition to presenting the integration findings in narrative format, authors should include joint displays that illustrate the researchers’ “cognitive process of merging, comparing, relating, and linking qualitative and quantitative data or results” (Guetterman et al., 2021). If the authors of the included studies had used these types of displays, integration would have been represented more clearly, making it easier for the reader to identify the meta-inferences (i.e., inferences derived from the integration of quantitative and qualitative findings in the form of theoretical statements, narratives, or a story) resulting from the MMR study.

### 4.3. Strengths and limitations

This study is a follow-up to a larger review of intervention studies aimed at increasing the participation of young people in STEM. Since the original review included all types of studies and no MMR-specific terms were used, we were able to accurately identify all MMR studies, including those that did not use this term (i.e., a total of 17 studies). In other words, we did not need to use method-specific terms because the initial sample included all relevant studies, including those that utilized MMR. The study had also some limitations. First, authors may use a wide variety of terms to describe the topic of the intervention, making it difficult to locate these types of studies in systematic reviews. Consequently, due to the search terms employed, it is possible that we overlooked several pertinent studies. Second, because MMR is still a developing methodology and some of its reporting components require further operationalization (e.g., evidence of integration), it is likely that authors of the included studies will disagree with some of our decisions during the extraction and coding processes. Third, we limited our evaluation of the quality of the included studies to the quality of the reporting and not the methodological quality. Future reviews could evaluate components of this other dimension of quality, such as whether the quantitative and qualitative components adhered to the quality criteria of each tradition or whether the divergences between the quantitative and qualitative findings have been adequately addressed (Hong et al., 2018, 2019).

## 5. Conclusion

In recent years, MMR has been widely utilized in intervention studies aimed at fostering an interest in STEM among young people. In these studies, researchers have incorporated qualitative research to overcome significant limitations of quantitative intervention designs to provide contextual knowledge easily transferable to practice. The included studies were generally adequately reported, particularly regarding the justification for adopting MMR and the integration of quantitative and qualitative data, two crucial components of MMR. However, some room for improvement was observed in a few components, namely, the description of the type of MMR design used, the explanation of the procedures in the qualitative component, and the use of joint displays for the systematic and visual representation of integration. More attention to these reporting standards will help ensure that the potential of MMR to provide a more comprehensive evaluation of the intervention is clearly communicated to readers.

## Data availability statement

The original contributions presented in this study are included in the article/Supplementary material, further inquiries can be directed to the corresponding authors.

## Author contributions

SF and MS: review conceptualization and design. SF: search strategy and writing—original draft. SF, MR, and B-SL-P: screening and study eligibility. SF, MS, MR, EE-B, and B-SL-P: data extraction and synthesis. All authors writing—review and editing and approved the submitted version.
