# Supplementary material for: Use of mixed methods research in intervention studies to increase young people’s interest in STEM: A systematic methodological review
Source: Front Psychol. 2023 Jan 5;13:956300. doi: 10.3389/fpsyg.2022.956300 (PMC9849589; doi:10.3389/fpsyg.2022.956300)
Supplement: Supplementary file 1 [file Data_Sheet_1.docx]

**Supplementary File 1**

*Search Strategy*

| Concept | Search terms (Title OR Abstract) |  |
| --- | --- | --- |
| Intervention | Program* OR Interven* OR Initiative* OR Strateg* OR Seminar* OR Workshop* OR Course* OR Session* | AND |
| STEM Studies and Professions | STEM OR Math* OR Science* OR Scient* OR Engineer* OR Technolog* OR "Technical stud*" OR career* OR "Technical career*" OR "Technical occupation*" OR "Technical subject*" OR "Scientific career*" OR "Scientific stud*" OR "Scientific occupation*" OR "Scientific subject*” | AND |
| Positive Outcomes | Interest* OR Engag* OR Motivat* OR Perform* OR Score* OR Grade* OR Abilit* OR Achiev* OR Choice* OR Selection OR Self- efficacy OR “Self-competence*" OR "Self-perception* of abilit*" OR "Sense of belonging" OR Stereotyp* OR Attitude* OR Participat* OR Involv* OR Capab* OR Encourag* OR Increas* OR Aspiration*OR “Self-concept*” | AND |
| Gender | Gender OR Girl* OR Female* OR Woman OR Women OR Sex |  |

Note. Search limits included English language and publication date between 1998 and 2019.

**Supplementary File 2**

*Data Extraction Form*

| **Publication metadata** |
| --- |
| Article ID |
| Publication year |
| First author name |
| First author affiliation |
| Article title |
| Journal name |

| **Study purpose** |
| --- |
| Study purpose |

| **Intervention characteristics** |
| --- |
| Country of the intervention |
| Geographical area of the intervention |
| Intervention name and description |
| STEM field |
| Main intervention outcomes |
| Type of intervention activity |
| Type of ordinary classroom activity (if applicable) |
| Type of extracurricular activity (if applicable) |
| Target intervention participants |
| Other intervention participants |
| Duration of the intervention |

| **Quantitative component** |
| --- |
| Purpose/research question |
| Time point assessed |
| Quantitative design/approach |
| Quantitative sampling strategy and participants |
| Quantitative data collection |
| Quantitative analysis |

| **Qualitative component** |
| --- |
| Purpose/research question |
| Time point assessed |
| Qualitative design/approach |
| Qualitative sampling strategy and participants |
| Qualitative data collection |
| Qualitative analysis |

| **Mixed methods research component** |
| --- |
| Terminology used to describe the mixed methods approach |
| Key mixed methods literature cited |
| Justification for using mixed methods research |
| Reported mixed methods research design |
| Point/Type of integration |
| Evidence of integration |
| Insights gained from integrating methods |
| Limitations of one method associated with the presence of the other method |

| **GRAMMS** |
| --- |
| Describes the justification for using a mixed methods approach to the research question |
| Describes the design in terms of the purpose, priority, and sequence of methods |
| Describes each method in terms of sampling, data collection and analysis |
| Reports evidence of integration |
| Describes any limitation of one method associated with the presence of the other method |
| Describes any insights gained from mixing or integrating methods |

**Supplementary File 3**

*Included Studies*

Aguilera, D. & Perales-Palacios, F. J. 2020. Learning biology and geology through a participative teaching approach: the effect on student attitudes towards science and academic performance. *J. Biol. Educ.*, 54, 245-261. 10.1080/00219266.2019.1569084.

Akkuş Çakır, N., Gass, A., Foster, A. & Lee, F. J. 2017. Development of a game-design workshop to promote young girls' interest towards computing through identity exploration. *Comput. Educ.*, 108, 115-130. 10.1016/j.compedu.2017.02.002.

Archer, L., Dewitt, J. & Dillon, J. 2014. 'It didn't really change my opinion': Exploring what works, what doesn't and why in a school science, technology, engineering and mathematics careers intervention. *Res. Sci. Technol. Educ.*, 32, 35-55. 10.1080/02635143.2013.865601.

Bamberger, Y. M. 2014. Encouraging girls into science and technology with feminine role model: Does this work? *J. Sci. Educ. Technol.*, 23, 549-561. 10.1007/s10956-014-9487-7.

Barak, M. & Asad, K. 2012. Teaching image-processing concepts in junior high school: Boys' and girls' achievements and attitudes towards technology. *Res. Sci. Technol. Educ.*, 30, 81-105. 10.1080/02635143.2012.656084.

Broder, E. D., Guilbert, K. E., Tinghitella, R. M., Murphy, S. M., Ghalambor, C. K. & Angeloni, L. M. 2019. Authentic science with dissemination increases self-efficacy of middle school students. *Integr. Comp. Biol.*, 59, 1497-1508. 10.1093/icb/icz140.

Chapman, A., Rodriguez, F. D., Pena, C., Hinojosa, E., Morales, L., Del Bosque, V., Tijerina, Y. & Tarawneh, C. 2020. “Nothing is impossible”: characteristics of Hispanic females participating in an informal STEM setting. *Cult. Stud. Sci. Educ.*, 15, 723-737. 10.1007/s11422-019-09947-6.

Clarke-Midura, J., Sun, C., Pantic, K., Poole, F. & Allan, V. 2019. Using informed design in informal computer science programs to increase youths' interest, self-efficacy, and perceptions of parental support. *ACM Trans. Comput. Educ.*, 1910.1145/3319445.

Collins, M. A., Totino, J., Hartry, A., Romero, V. F., Pedroso, R. & Nava, R. 2020. Service-Learning as a Lever to Support STEM Engagement for Underrepresented Youth. *J. Exp. Educ.*, 43, 55-70. 10.1177/1053825919887407.

Denner, J., Werner, L., Martinez, J. & Bean, S. 2012. Computing goals, values, and expectations: Results from an after-school program for girls. J. Women Minor. Sci. Eng., 18, 199-213. 10.1615/JWomenMinorScienEng.2013002075.

Fabian, K. & Topping, K. J. 2019. Putting “mobile” into mathematics: Results of a randomised controlled trial. *Contemp. Educ. Psychol.*, 5910.1016/j.cedpsych.2019.101783.

Ferreira, M. 2002. Ameliorating equity in science, mathematics, and engineering: A case study of an after-school science program. *Equity Excell. Educ.*, 35, 43-49. 10.1080/713845242.

Grover, S., Jackiw, N. & Lundh, P. 2019. Concepts before coding: non-programming interactives to advance learning of introductory programming concepts in middle school. *Comput. Sci. Educ.*, 29, 106-135. 10.1080/08993408.2019.1568955.

Hughes, R. M., Nzekwe, B. & Molyneaux, K. J. 2013. The Single Sex Debate for Girls in Science: A Comparison Between Two Informal Science Programs on Middle School Students' STEM Identity Formation. *Res. Sci. Educ.*, 43, 1979-2007. 10.1007/s11165-012-9345-7.

Hur, J. W., Andrzejewski, C. E. & Marghitu, D. 2017. Girls and computer science: experiences, perceptions, and career aspirations. *Comp. Sci. Educ.*, 27, 100-120. 10.1080/08993408.2017.1376385.

Kebritchi, M., Hirumi, A. & Bai, H. 2010. The effects of modern mathematics computer games on mathematics achievement and class motivation. *Comp. Educ.*, 55, 427-443. 10.1016/j.compedu.2010.02.007.

Ketelhut, D. J., Nelson, B. C., Clarke, J. & Dede, C. 2010. A multi-user virtual environment for building and assessing higher order inquiry skills in science. *Br. J. Educ. Technol.*, 41, 56-68. 10.1111/j.1467-8535.2009.01036.x.

Kim, H. 2011. Inquiry-Based Science and Technology Enrichment Program: Green Earth Enhanced with Inquiry and Technology. *J. Sci. Educ. Technol.*, 20, 803-814. 10.1007/s10956-011-9334-z.

Kim, H. 2016. Inquiry-Based Science and Technology Enrichment Program for Middle School-Aged Female Students. *J. Sci. Educ. Technol.*, 25, 174-186. 10.1007/s10956-015-9584-2.

Koch, M., Georges, A., Gorges, T. & Fujii, R. 2010. Engaging youth with STEM professionals in afterschool programs. *Meridian Middle School Comp. Technol. J.*, 13, 1-15.

Lang, C., Fisher, J., Craig, A. & Forgasz, H. 2015. Outreach programmes to attract girls into computing: how the best laid plans can sometimes fail. *Comp. Sci. Educ.*, 25, 257-275. 10.1080/08993408.2015.1067008.

Lin, Y. T., Wang, M. T. & Wu, C. C. 2019. Design and Implementation of Interdisciplinary STEM Instruction: Teaching Programming by Computational Physics. *Asia-Pac. Educ. Res.*, 28, 77-91. 10.1007/s40299-018-0415-0.

Magerko, B., Freeman, J., Mcklin, T., Reilly, M., Livingston, E., Mccoid, S. & Crews-Brown, A. 2016. EarSketch: A STEAM-based approach for underrepresented populations in high school computer science education. *ACM Trans. Comput. Educ.*, 1610.1145/2886418.

Marino, M. T., Israel, M., Beecher, C. C. & Basham, J. D. 2013. Students' and Teachers' Perceptions of Using Video Games to Enhance Science Instruction. *J. Sci. Educ. Technol.*, 22, 667-680. 10.1007/s10956-012-9421-9.

Mavridis, A., Katmada, A. & Tsiatsos, T. 2017. Impact of online flexible games on students’ attitude towards mathematics. *Educ. Tech. Res. Dev.*, 65, 1451-1470. 10.1007/s11423-017-9522-5.

Munson, A. M., Harralson, A. F., Plack, M. M. & Nickola, T. J. 2014. Out-of-school genomics program for young women demystifies genomics and fosters interest in biomedical sciences. *Pharmacogenomics*, 15, 265-276. 10.2217/pgs.14.5.

Ogle, J. P., Hyllegard, K. H., Rambo-Hernandez, K. & Park, J. 2017. Building middle school girls’ self-efficacy, knowledge, and interest in math and science through the integration of fashion and STEM. *J. Fam. Cons. Sci.*, 109, 33-40.

Pedersen, S. & Williams, D. 2004. A comparison of assessment practices and their effects on learning and motivation in a student-centered learning environment. *J. Educ. Multimed. Hypermed.*, 13, 283-306.

Ruth, A., Hackman, J., Brewis, A., Spence, T., Luchmun, R., Velez, J. & Ganesh, T. G. 2019. Engineering projects in community service (Epics) in high schools: Subtle but potentially important student gains detected from human-centered curriculum design. *Educ. Sci.*, 910.3390/educsci9010035.

Simon, U. K., Steindl, H., Larcher, N., Kulac, H. & Hotter, A. 2016. Young science journalism: Writing popular scientific articles may contribute to an increase of high-school students’ interest in the natural sciences. *Int. J. Sci. Educ.*, 38, 814-841. 10.1080/09500693.2016.1173260.

Skipper, Y. & De Carvalho, E. 2019. “I Have Seen the Opportunities That Science Brings”: Encouraging Girls to Persist in Science. *Educ. Forum*, 83, 199-214. 10.1080/00131725.2019.1576820.

Solís, P., Huynh, N. T., Huot, P., Zeballos, M., Ng, A. & Menkiti, N. 2019. Towards an overdetermined design for informal high school girls’ learning in geospatial technologies for climate change. *Int. Res. Geogr. Env. Educ.*, 28, 151-174. 10.1080/10382046.2018.1513447.

Stake, J. E. & Mares, K. R. 2005. Evaluating the impact of science-enrichment programs on adolescents' science motivation and confidence: The splashdown effect. *J. Res. Sci. Teach.*, 42, 359-375. 10.1002/tea.20052.

Todd, B. & Zvoch, K. 2019. Exploring Girls’ Science Affinities Through an Informal Science Education Program. *Res. Sci. Educ.*, 49, 1647-1676. 10.1007/s11165-017-9670-y.

**Supplementary File 4**

*Characteristics of the Included Studies in the Review (n = 34)*

| **Study (year)^1^** | **Journal name** | **Country of the intervention** | **Intervention name** | **Field** | **Main intervention outcomes** | **Sample** | **Theory mentioned** | **QUAN research purposes** | **QUAL research purposes** |
| --- | --- | --- | --- | --- | --- | --- | --- | --- | --- |
| Aguilera & Perales-Palacios (2020) | Journal of Biological Education | Spain | Participative Teaching Approach Intervention | Science | Achievement, Motivation | 28 girls, 29 boys, 1 teacher | No | Effectiveness | Effectiveness |
| Akkuş Çakır et al. (2017) | Computers & Education | USA | Game-Design Workshop Through Identity Exploration | Technology | Motivation, Identity | 21 girls | Yes | Effectiveness | Effectiveness, acceptability |
| Archer et al. (2014) | Research in Science & Technological Education | UK | The ASPIRES Project: A Pilot STEM Careers Intervention | STEM | Motivation, Identity | 68 girls, 1 teacher | No | Effectiveness | Effectiveness, acceptability, fidelity |
| Bamberger (2014) | Journal of Science Education and Technology | Israel | BAMOT Project | STEM | Motivation, Gender stereotypes, Study Choices | 90 girls | Yes | Effectiveness | Effectiveness |
| Barak & Asad (2012) | Research in Science & Technological Education | Israel | Course on Image-Processing Principles | STEM | Achievement, Motivation | 31 girls, 29 boys | Yes | Effectiveness | Effectiveness |
| Broder et al. (2019) | Integrative and Comparative Biology | USA | "Science Club": Science After-School Program | STEM | Motivation | 9 girls, 23 boys | Yes | Effectiveness | Effectiveness |
| Chapman et al. (2020) | Cultural Studies of Science Education | USA | Railway Safety Summer Camp | STEM | Achievement | 171 girls, 263 boys | No | Effectiveness | Effectiveness |
| Clarke-Midura et al. (2019) | ACM Transactions on Computing Education | USA | App Camp | Technology | Motivation, Emotional | 71 girls, 40 boys | Yes | Effectiveness | Effectiveness |
| Collins et al. (2020) | Journal of Experiential Education | USA | EPICC Summer Program | STEM | Achievement, Motivation | 49 girls, 43 boys | Yes | Effectiveness | Effectiveness, acceptability |
| Denner et al. (2012) | Journal of Women and Minorities in Science and Engineering | USA | Girl Game Company Program | Technology | Motivation, Gender stereotypes | 59 girls | Yes | Effectiveness | Effectiveness |
| Fabian & Topping (2019) | Contemporary Educational Psychology | UK | Mobile Learning in Mathematics | Science | Achievement, Motivation | 37 girls, 37 boys, 3 teachers | Yes | Effectiveness | Effectiveness, acceptability, feasibility |
| Ferreira (2002) | Equity & Excellence in Education | USA | After-School Science Program | STEM | Motivation, Gender stereotypes | 18 girls, 7 engineers | No | Effectiveness | Effectiveness, acceptability |

Supplementary File 4 Continued

| **Study (year)^1^** | **Journal name** | **Country of the intervention** | **Intervention name** | **Field** | **Main intervention outcomes** | **Sample** | **Theory mentioned** | **QUAN research purposes** | **QUAL research purposes** |
| --- | --- | --- | --- | --- | --- | --- | --- | --- | --- |
| Grover et al. (2019) | Computer Science Education | USA | VELA Project | Technology | Achievement, Motivation | 28 girls, 43 boys, 3 teachers | Yes | Effectiveness | Effectiveness |
| Hughes et al. (2013) | Research in Science Education | USA | Cultivating Opportunities in Engineering Disciplines & Getting Involved in Research and Learning in Science summer camps | STEM | Motivation, Identity | 45 girls, 14 boys, teachers | Yes | Effectiveness | Effectiveness |
| Hur et al. (2017) | Computer Science Education | USA | Computer Science for All Girls Camp (CS4ALL-G) | Technology | Motivation | 27 girls | No | Effectiveness, acceptability | Effectiveness, acceptability |
| Kebritchi et al. (2010) | Computers & Education | USA | DimensionM | Science | Achievement, Motivation | 102 girls, 91 boys, 10 teachers | Yes | Effectiveness | Effectiveness |
| Ketelhut et al. (2010) | British Journal of Educational Technology | USA | River City Virtual Environment | Science | Achievement, Motivation | 2000 girls and boys (approx.), teachers | No | Effectiveness | Effectiveness, acceptability |
| Kim (2011) | Journal of Science Education and Technology | USA | InSTEP Inquiry-Based Science and Technology Enrichment Program | STEM | Achievement, Motivation, Gender stereotypes, Emotional | 35 girls | No | Effectiveness | Effectiveness |
| Kim (2016) | Journal of Science Education and Technology | USA | InSTEP Inquiry-Based Science and Technology Enrichment Program | Science | Achievement, Motivation, Gender stereotypes | 123 girls | No | Effectiveness | Effectiveness |
| Koch et al. (2010) | Meridian Middle School Computer Technologies Journal | USA, Canada | Build IT | Technology | Motivation, Gender stereotypes | 439 girls, 21 professionals | No | Effectiveness | Effectiveness, fidelity |
| Lang et al. (2015) | Computer Science Education | Australia | Digital Divas Club | Technology | Motivation, Gender stereotypes | 265 girls, 18 teachers | No | Effectiveness, acceptability | Effectiveness, acceptability |
| Lin et al. (2019) | The Asia-Pacific Education Researcher | Taiwan | Interdisciplinary STEM Instruction: Teaching Programming by Computational Physics | STEM | Achievement, Motivation | 89 girls, 60 boys | Yes | Effectiveness | Effectiveness |
| Magerko et al. (2016) | ACM Transactions on Computing Education | USA | EarSketch | STEAM | Achievement, Motivation, Identity | 26 girls, 71 boys | Yes | Effectiveness | Effectiveness |

Supplementary File 4 Continued

| **Study (year)^1^** | **Journal name** | **Country of the intervention** | **Intervention name** | **Field** | **Main intervention outcomes** | **Sample** | **Theory mentioned** | **QUAN research purposes** | **QUAL research purposes** |
| --- | --- | --- | --- | --- | --- | --- | --- | --- | --- |
| Marino et al. (2013) | Journal of Science Education and Technology | USA | Video Games to Enhance Science Instruction | STEAM | Motivation | 429 girls, 447 boys, 34 teachers | No | Effectiveness | Effectiveness, acceptability, feasibility |
| Mavridis et al. (2017) | Educational Technology Research & Development | Greece | Online flexible educational game | Science | Achievement, Motivation | 33 girls, 46 boys, 4 teachers | No | Effectiveness | Effectiveness, feasibility |
| Munson et al. (2014) | Pharmacogenomics | USA | Genomics Opportunities for Girls in Research Labs (GO GIRL) | Science | Achievement, Motivation | 65 girls | No | Effectiveness | Effectiveness, acceptability |
| Ogle et al. (2017) | Journal of Family & Consumer Sciences | USA | Fashion FUNdamentals (FF) | Science | Achievement, Motivation | 52 girls | No | Effectiveness | Effectiveness |
| Pedersen & Williams (2004) | Journal of Educational Multimedia and Hypermedia | USA | Alien Rescue | Science | Achievement, Motivation | 77 girls and boys | Yes | Effectiveness | Effectiveness |
| Ruth et al. (2019) | Education Sciences | USA | Engineering Projects in Community Service (EPICS) High | Technology | Motivation | 111 girls, 147 boys | Yes | Effectiveness | Effectiveness |
| Simon et al. (2016) | International Journal of Science Education | Austria | Young Science Journalism | STEAM | Motivation | 7 girls, 13 boys | Yes | Effectiveness | Effectiveness |
| Skipper & de Carvalho (2019) | The Educational Forum | UK | Interactive Science Sessions on the Women in Science Day | Science | Motivation | 66 girls | Yes | Effectiveness | Effectiveness |
| Solís et al. (2019) | International Research in Geographical and Environmental Education | Bolivia, South Africa, Panama | Global Connections and Exchange - My Community, Our Earth Youth TechCamps | STEM | Motivation | 66 girls, 50 boys | No | Effectiveness | Effectiveness |
| Stake & Mares (2005) | Journal of Research in Science Teaching | USA | Science Enrichment Program | Science | Motivation, Identity | 47 girls, 41 boys | Yes | Effectiveness | Effectiveness |
| Todd & Zvoch (2019) | Research in Science Education | USA | Science Outreach Program and Summer Camps for Girls | STEM | Motivation, Identity, Gender stereotypes | 55 girls | Yes | Effectiveness | Effectiveness, acceptability, fidelity |

^1^Studies are presented in alphabetical order. The full citation for each study can be found in Supplementary File 3.

^2^While the authors state that the study was conducted in a “primary” school, we included it because some of the participants were in grade 6 (consistent with our inclusion criteria).

Note: QUAN=Quantitative, QUAL=Qualitative, STEM=Science, Technology, Engineering, and Mathematics, STEAM=Science, Technology, Engineering, Arts, and Mathematics.

**Supplementary File 5**

*Cited Key Literature on Mixed Methods Research*

| **Bibliographic information** | **N**^1^ | **%** |
| --- | --- | --- |
| Creswell, J. W., & Plano Clark, V. L. (2007). *Designing and conducting mixed methods research*. Sage. | 4 | 29 |
| Creswell, J. W. (2003). *Research design: Qualitative, quantitative, and mixed methods approaches* (2nd ed.). Sage. | 2 | 14 |
| Bryman, A. (2007). Barriers to integrating quantitative and qualitative research. *Journal of Mixed Methods Research, 1*(1), 8-22. <https://doi.org/10.1177/2345678906290531> | 1 | 7 |
| Creswell, J. W. (2012). *Educational research: Planning, conducting, and evaluating quantitative and qualitative research* (4th ed.). Pearson. | 1 | 7 |
| Creswell, J. W. (2014). *Research design: Qualitative, quantitative, and mixed methods approaches* (4th ed.). Sage. | 1 | 7 |
| Creswell, J. W., & Plano Clark, V. L. (2010). *Designing and conducting mixed methods research* (2nd ed.). Sage. | 1 | 7 |
| Greene, J. C. (2005). The generative potential of mixed methods inquiry. *International Journal of Research and Method in Education, 28*(2), 207-211. <https://doi.org/10.1080/01406720500256293> | 1 | 7 |
| Hesse-Biber, S., & Johnson, R. B. (2013). Coming at things differently: Future directions of possible engagement with mixed methods research. *Journal of Mixed Methods Research, 7*(2), 103-109. <https://doi.org/10.1177/1558689813483987> | 1 | 7 |
| Leech, N. L., & Onwuegbuzie, A. J. (2009). A typology of mixed methods research designs. *Quality and Quantity, 43*(2), 265-275. <https://doi.org/10.1007/s11135-007-9105-3> | 1 | 7 |
| Venkatesh, V., Brown, S. A., & Bala, H. (2013). Bridging the qualitative-quantitative divide: Guidelines for conducting mixed methods research in information systems. *MIS Quarterly: Management Information Systems, 37*(1), 21-54. <https://doi.org/10.25300/MISQ/2013/3> | 1 | 7 |

^1^Number of times the reference has been cited. One study could cite more than one reference.

**Supplementary File 6**

*Summary of Mixed Methods Features of the Included Studies (n = 34)*

| **Study (year)^1^** | **MMR rationale** | **Reported mixed methods design** | **QUAN design and methods** | **QUAL design and methods** | **Stage of QUAL research with respect to the intervention** | **Integration at the methods level** | **Integration at the interpretation/ reporting level** | **Description of the MMR insights gained** |
| --- | --- | --- | --- | --- | --- | --- | --- | --- |
| Aguilera & Perales-Palacios (2020) | Explicit | Convergent design | SGPPTD, questionnaire, observation checklist, school records | Ethnographic design, interviews | After | Merging | Narrative | Partial |
| Akkuş Çakır et al. (2017) | Explicit | NR^2^ | SGPPTD, questionnaire, quantitative content analysis of games | Focus groups | After | Merging | Narrative | Explicit |
| Archer et al. (2014) | Explicit | NR^2^ | SGPPTD, questionnaire | Interviews, focus groups, observation, open-ended questions | Before, during, after, and follow-up | Merging | Narrative | Explicit |
| Bamberger (2014) | Partial | NR^2^ | MGPPTD, questionnaire | Focus groups, observation, open-ended questions | Before, during, and after | Merging | Narrative, Data transformation | NR |
| Barak & Asad (2012) | Explicit | NR^2^ | SGPPTD, questionnaire, achievement exam | Observation, open-ended questions | Before, during, and after | Merging | Narrative | NR |
| Broder et al. (2019) | Explicit | Explanatory sequential | SGPPTD, questionnaire | Interviews | After | Merging | Narrative | Explicit |
| Chapman et al. (2020) | Explicit | Convergent design | SGPPTD, questionnaire | Interviews | After | Merging | Narrative | NR |
| Clarke-Midura et al. (2019) | Explicit | NR^2^ | SGPPTD, questionnaire | Interviews | After | Merging | Narrative | Partial |
| Collins et al. (2020) | Partial | NR^2^ | MGPPTD (retrospective), questionnaire, observation checklist | Open-ended questions | After | Merging | Narrative | NR |
| Denner et al. (2012) | Explicit | NR^2^ | SGPPTD, questionnaire | Open-ended questions, drawings | Before and after | Merging | Narrative | NR |
| Fabian & Topping (2019) | Explicit | NR^2^ | RCT, questionnaire, achievement exam | Interviews, group interviews | After | Merging | Narrative | Explicit |
| Ferreira (2002) | Partial | NR^2^ | SGPPTD, questionnaire | Interviews | After | Merging | Narrative | NR |

Supplementary File 6 Continued

| **Study (year)^1^** | **MMR rationale** | **Reported mixed methods design** | **QUAN design and methods** | **QUAL design and methods** | **Stage of QUAL research with respect to the intervention** | **Integration at the methods level** | **Integration at the interpretation/ reporting level** | **Description of the MMR insights gained** |
| --- | --- | --- | --- | --- | --- | --- | --- | --- |
| Grover et al. (2019) | Partial | NR^2^ | SGPPTD, questionnaire, evaluation rubric | Interviews, focus groups | During and after | Merging | Narrative | NR |
| Hughes et al. (2013) | Explicit | NR^2^ | SGPPTD, questionnaire | Interviews, observation, open-ended questions | Before, during, and after | Merging, connecting | Narrative | NR |
| Hur et al. (2017) | Explicit | Convergent design | SGPPTD, questionnaire | Focus groups, participant observation, open-ended questions | During and after | Merging | Narrative | NR |
| Kebritchi et al. (2010) | Explicit | NR^2^ | MGPPTD, questionnaire, achievement exam | Interviews | After | Merging | Narrative | Explicit |
| Ketelhut et al. (2010) | Partial | NR^2^ | RCT, questionnaire, quantitative content analysis of letters | Interviews | Before, during, and after | Merging | Narrative | NR |
| Kim (2011) | Partial | NR^2^ | SGPPTD, questionnaire, content knowledge test | Interviews, open-ended questions | After | Merging | Narrative | Partial |
| Kim (2016) | Partial | NR^2^ | SGPPTD, questionnaire, content knowledge test | Interviews, open-ended questions, drawings | After | Merging | Narrative | NR |
| Koch et al. (2010) | Explicit | NR^2^ | MGPPTD, questionnaire | Interviews, observation, feedback forms | Before, during, and after | Merging | Narrative | NR |
| Lang et al. (2015) | Explicit | NR^2^ | SGPPTD, questionnaire | Interviews, focus groups, open-ended questions | Before, after, and follow-up | NEI | NEI | NR |
| Lin et al. (2019) | Explicit | NR | MGPPTD, questionnaire, achievement exam | Interviews, student worksheets | NR | Merging | Narrative | NR |
| Magerko et al. (2016) | Explicit | NR^2^ | SGPPTD (retrospective), questionnaire, content knowledge test | Focus groups | After | Merging, building | Narrative | NR |

Supplementary File 6 Continued

| **Study (year)^1^** | **MMR rationale** | **Reported mixed methods design** | **QUAN design and methods** | **QUAL design and methods** | **Stage of QUAL research with respect to the intervention** | **Integration at the methods level** | **Integration at the interpretation/ reporting level** | **Description of the MMR insights gained** |
| --- | --- | --- | --- | --- | --- | --- | --- | --- |
| Marino et al. (2013) | Partial | NR^2^ | SGPPTD, questionnaire | Interviews | After | Merging, building | Narrative | NR |
| Mavridis et al. (2017) | Explicit | NR^2^ | RCT, questionnaire, achievement exam | Interviews | After | NEI | NEI | NR |
| Munson et al. (2014) | Partial | NR^2^ | SGPPTD, questionnaire | Focus groups | Follow-up | Merging | Narrative | NR |
| Ogle et al. (2017) | Partial | NR^2^ | SGPPTD, questionnaire | Focus groups | After | Merging | Narrative | NR |
| Pedersen & Williams (2004) | Partial | NR^2^ | RCT, questionnaire, factual knowledge test, problem solutions, artifacts, worksheets | Interviews | After | Merging | Narrative | NR |
| Ruth et al. (2019) | Partial | NR^2^ | SGPPTD, questionnaire | Open-ended questions, interviews, observation | Before, during, and after | Merging | Narrative | Explicit |
| Simon et al. (2016) | Partial | NR^2^ | SGPPTD, questionnaire | Interviews, open-ended questions | Before and after | Merging | Narrative | NR |
| Skipper & de Carvalho (2019) | Explicit | NR^2^ | SGPPTD, questionnaire | Open-ended questions | Before and after | Merging | Narrative | NR |
| Solís et al. (2019) | Explicit | NR^2^ | SGPPTD, questionnaire | Open-ended questions | Before, after, and follow-up | Merging | Narrative | NR |
| Stake & Mares (2005) | Partial | NR^2^ | SGPPTD, questionnaire | Interviews | Follow-up | Merging | Narrative | NR |
| Todd & Zvoch (2019) | Partial | NR^2^ | SGPPTD, questionnaire | Interviews, focus groups, observation, fidelity rubric | During and follow-up | NEI | NEI | NR |

^1^Studies are presented in alphabetical order.

^2^Studies that provide a precise description of the strand timing but do not report the type of design.

Note. QUAN=Quantitative, QUAL=Qualitative, MMR=Mixed methods research, RCT=Randomized controlled trial, SGPPTD=Single group pre-post treatment design, MGPPTD=Multiple group pre-post treatment design, NEI=Not evidence of integration, NR=Not reported

**Supplementary File 7**

*Description of the Adapted Good Reporting of a Mixed Methods Study (GRAMMS) Guidelines*

| **GRAMMS Guideline** | **Yes** | **Yes, but** | **No** |
| --- | --- | --- | --- |
| 1. Describes the justification for using a mixed methods approach to the research question | Provides an explicit justification for using mixed methods research in the introduction or the methods section. | Does not provide an explicit justification for using mixed methods research in the introduction or the methods section, but this justification can be inferred. | Does not provide an explicit justification for using mixed methods research in the introduction or the methods section, and this justification cannot be inferred. |
| 1. Describes the mixed methods design in terms of the purpose, priority, and sequence of methods | Provides a name for the type of mixed methods research design used and describes the priority and/or the timing of the quantitative and qualitative components (usually reported in the methods section). | Does not provide a name for the type of mixed methods research design used but describes the priority and/or the timing of the components (usually reported in the methods section). | Does not provide a name for the type of mixed methods research design nor describe priority and/or timing of the components (usually reported in the methods section). |
| 1. Describes each method in terms of sampling, data collection and analysis | Describes all (six) the elements of the quantitative and qualitative components (i.e., sampling/participants selection, type of data sources, type of analyses) (usually reported in the methods section). | Describes four or five elements of the quantitative and qualitative components (i.e., sampling/participants selection, type of data sources, type of analyses) (usually reported in the methods section). | Describes less than four elements of the quantitativ and qualitative components (i.e., sampling/participants selection, type of data sources, type of analyses) (usually reported in the methods section). |
| 1. Reports evidence of integration | Reports explicit evidence of integration in the Results or Discussion/Conclusion sections in one of these forms: joint displays, data transformation, or narrative. | Does not report explicit evidence of integration, but the integration outcomes can be inferred. | Does not report any evidence of integration and the integration outcomes cannot be inferred. |
| 1. Describes any limitation of one method associated with the presence of the other method | Provides an explicit description of any limitation of one method associated with the presence of the other method (usually reported in the discussion/conclusion section). | Does not provide an explicit description of any limitation of one method associated with the presence of the other method, but this limitation can be inferred (usually reported in the discussion/conclusion section). | Does not provide an explicit description of any limitation of one method associated with the presence of the other method, and this limitation cannot be inferred (usually reported in the discussion/conclusion section). |
| 1. Describes any insights gained from mixing or integrating methods | Provides an explicit description of the insights gained from mixing or integrating methods in the discussion/conclusion section. | Does not provide an explicit description of the insights, but these insights can be inferred in the discussion/conclusion section. | Does not provide an explicit description of the insights and these insights cannot be inferred in the discussion/conclusion section. |
